# Evaporative resistance of newly designed bicycle helmets

**DOI:** 10.1186/2046-7648-4-S1-A72

**Published:** 2015-09-14

**Authors:** Kalev Kuklane, Helena Aljaste, Sixten S Heidmets, COST Action TU1101 WG4

**Affiliations:** 1 The Thermal Environment Laboratory, Division of Ergonomics and Aerosol Technology, Department of Design Sciences, Faculty of Engineering, Lund University, Lund, Sweden; 2 Department of Product Design, Estonian Academy of Arts, Tallinn, Estonia; 3 HOPE-Helmet OPtimization in Europe, COST Action TU1101 Working Group 4 with the participation of Jean-Marie Aerts, Helena Aljaste, Simon Annaheim, Cornelis P Bogerd, Peter Bröde, Guido De Bruyne, Andreas D Flouris, Sixten S Heidmets, Anica Hursa, Kalev Kuklane, Tiago S Mayor, René M Rossi

## Introduction

As a continuation of the work on the ventilation requirements for a bicycle helmet for commuters [1], 15 full scale helmet mock-ups were created and tested for dry heat loss properties [2] in a wind tunnel on a thermal head manikin [3]. This paper presents the results of the wet heat transfer measurements in the form of evaporative resistance.

## Methods

The helmets were tested in the wind tunnel placed in a climatic chamber at 34 °C and 40 % relative humidity (water vapour pressure in the air 2200 Pa) with the air velocities set to 1.6 m/s (≈6 km/h). Tests were performed on the head model without a wig. Evaporative resistance was calculated from heat loss corrected for the difference in head surface to textile skin temperature [4]. One (TK) of the 15 mock-ups was tested back to front, too (TKo). In addition, 4 commercially available helmets were tested as reference. Three of them were one of the best, one average and one of the less well performing helmets of the study by Brühwiler et al. [5], and one was a helmet often bought and used by commuters.

## Results

**Figure 1:**
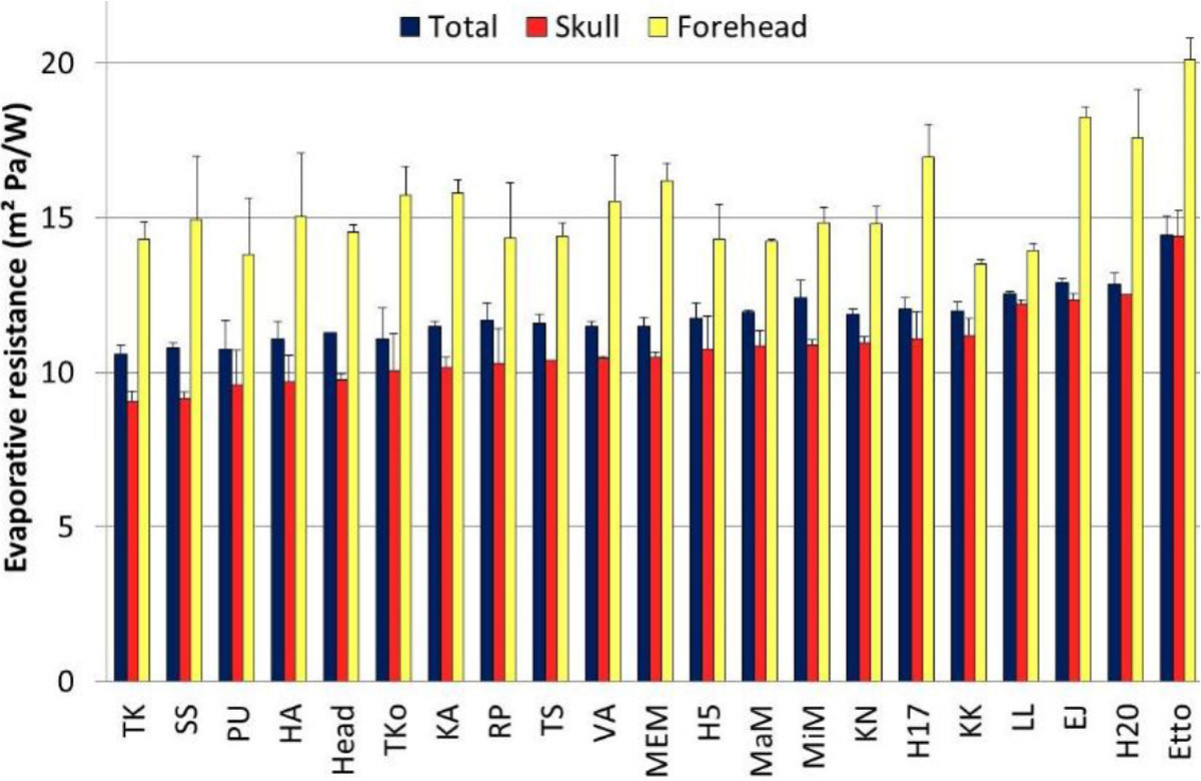
Evaporative resistance of design helmets at 1.6 m.s^-1 ^wind and 34 °C with a wet textile skin

The prototypes differed largely in evaporative resistance (Figure 1).

## Discussion

The best reference helmet from the earlier study [5] stayed approximately in the middle of the tested range while the poorest reference helmet was in the end of the line with an often used one as the worst (Figure 1). Thus, the newer solutions performed much better from an evaporation viewpoint than commercially available helmets. However, the order of the helmets in the "best function line" for evaporation was different from their insulation performance, as reported previously [2].

## Conclusion

The best helmet from the evaporation viewpoint was different from the best in terms of insulation. This means that a best solution for a commuter has to be defined by the user's bicycling activity, the weather conditions etc. The newly designed helmets' results can be used as the basis for improvement of helmet ventilation.
